# Supplementary material for: Towards a structurally resolved human protein interaction network
Source: Nat Struct Mol Biol. 2023 Jan 23;30(2):216–25. doi: 10.1038/s41594-022-00910-8 (PMC9935395; doi:10.1038/s41594-022-00910-8)
Supplement: Supplementary file 1 — Supplementary Figs. 1–4. [file 41594_2022_910_MOESM1_ESM.pdf]

nature structural & molecular biology

Article
https://doi.org/10.1038/s41594-022-00910-8

Towards a structurally resolved human protein interaction network

In the format provided by the authors and unedited

Figure S1:
All the strongest up- or down-regulation phosphosites for each group.

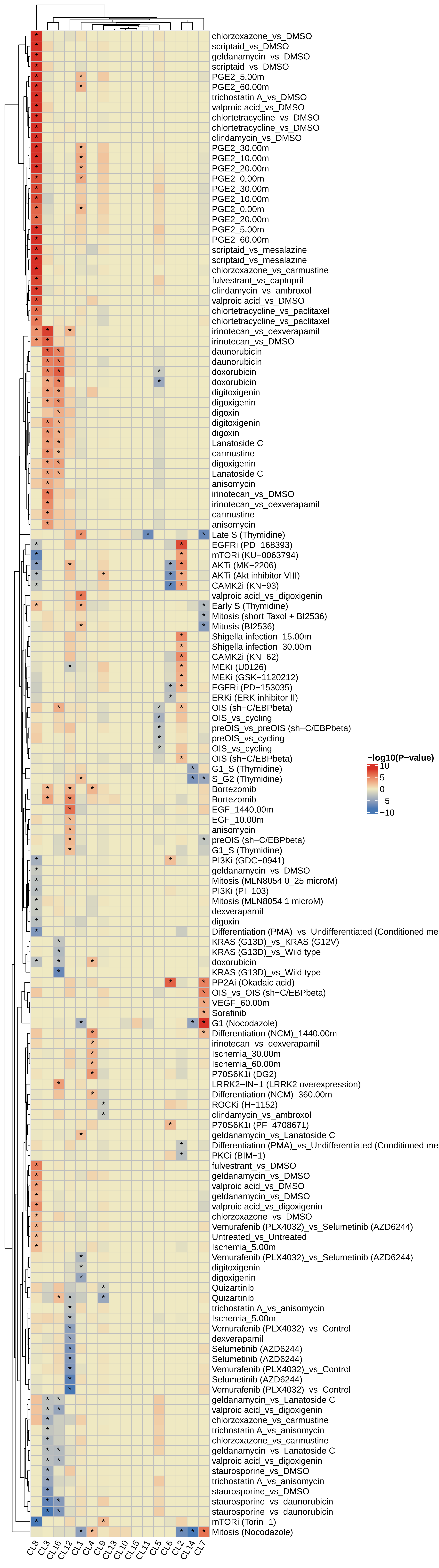
chlorzoxazone_vs_DMSO
scriptaid_vs_DMSO
geldanamycin_vs_DMSO
scriptaid_vs_DMSO
PGE2_5.00m
PGE2_60.00m
trichostatin A_vs_DMSO
valproic acid_vs_DMSO
chlortetracycline_vs_DMSO
chlortetracycline_vs_DMSO
clindamycin_vs_DMSO
PGE2_30.00m
PGE2_10.00m
PGE2_20.00m
PGE2_0.00m
PGE2_30.00m
PGE2_10.00m
PGE2_0.00m
PGE2_20.00m
PGE2_5.00m
PGE2_60.00m
scriptaid_vs_mesalazine
scriptaid_vs_mesalazine
chlorzoxazone_vs_carmustine
fulvestrant_vs_captopril
clindamycin_vs_ambroxol
valproic acid_vs_DMSO
chlortetracycline_vs_paclitaxel
chlortetracycline_vs_paclitaxel
irinotecan_vs_dexverapamil
irinotecan_vs_DMSO
daunorubicin
daunorubicin
doxorubicin
doxorubicin
digitoxigenin
digoxigenin
digoxin
digitoxigenin
digoxin
Lanatoside C
carmustine
digoxigenin
Lanatoside C
anisomycin
irinotecan_vs_DMSO
irinotecan_vs_dexverapamil
carmustine
anisomycin
Late S (Thymidine)
EGFRi (PD−168393)
mTORi (KU−0063794)
AKTi (MK−2206)
AKTi (Akt inhibitor VIII)
CAMK2i (KN−93)
valproic acid_vs_digoxigenin
Early S (Thymidine)
Mitosis (short Taxol + BI2536)
Mitosis (BI2536)
Shigella infection_15.00m
Shigella infection_30.00m
CAMK2i (KN−62)
MEKi (U0126)
MEKi (GSK−1120212)
EGFRi (PD−153035)
ERKi (ERK inhibitor II)
OIS (sh−C/EBPbeta)
OIS_vs_cycling
preOIS_vs_preOIS (sh−C/EBPbeta)
preOIS_vs_cycling
OIS_vs_cycling
OIS (sh−C/EBPbeta)
G1_S (Thymidine)
S_G2 (Thymidine)
Bortezomib
Bortezomib
EGF_1440.00m
EGF_10.00m
anisomycin
preOIS (sh−C/EBPbeta)
G1_S (Thymidine)
PI3Ki (GDC−0941)
geldanamycin_vs_DMSO
Mitosis (MLN8054 0_25 microM)
PI3Ki (PI−103)
Mitosis (MLN8054 1 microM)
dexverapamil
digoxin
Differentiation (PMA)_vs_Undifferentiated (Conditioned me
KRAS (G13D)_vs_KRAS (G12V)
KRAS (G13D)_vs_Wild type
doxorubicin
KRAS (G13D)_vs_Wild type
PP2Ai (Okadaic acid)
OIS_vs_OIS (sh−C/EBPbeta)
VEGF_60.00m
Sorafinib
G1 (Nocodazole)
Differentiation (NCM)_1440.00m
irinotecan_vs_dexverapamil
Ischemia_30.00m
Ischemia_60.00m
P70S6K1i (DG2)
LRRK2−IN−1 (LRRK2 overexpression)
Differentiation (NCM)_360.00m
ROCKi (H−1152)
clindamycin_vs_ambroxol
P70S6K1i (PF−4708671)
geldanamycin_vs_Lanatoside C
Differentiation (PMA)_vs_Undifferentiated (Conditioned me
PKCi (BIM−1)
fulvestrant_vs_DMSO
geldanamycin_vs_DMSO
valproic acid_vs_DMSO
geldanamycin_vs_DMSO
valproic acid_vs_digoxigenin
chlorzoxazone_vs_DMSO
Vemurafenib (PLX4032)_vs_Selumetinib (AZD6244)
Untreated_vs_Untreated
Ischemia_5.00m
Vemurafenib (PLX4032)_vs_Selumetinib (AZD6244)
digitoxigenin
digoxigenin
Quizartinib
Quizartinib
trichostatin A_vs_anisomycin
Ischemia_5.00m
Vemurafenib (PLX4032)_vs_Control
dexverapamil
Selumetinib (AZD6244)
Selumetinib (AZD6244)
Vemurafenib (PLX4032)_vs_Control
Selumetinib (AZD6244)
Vemurafenib (PLX4032)_vs_Control
geldanamycin_vs_Lanatoside C
valproic acid_vs_digoxigenin
chlorzoxazone_vs_carmustine
trichostatin A_vs_anisomycin
chlorzoxazone_vs_carmustine
geldanamycin_vs_Lanatoside C
valproic acid_vs_digoxigenin
staurosporine_vs_DMSO
trichostatin A_vs_anisomycin
staurosporine_vs_DMSO
staurosporine_vs_daunorubicin
staurosporine_vs_daunorubicin
mTORi (Torin−1)
Mitosis (Nocodazole)
CL8
CL3
CL16
CL12
CL1
CL4
CL9
CL13
CL10
CL15
CL11
CL5
CL6
CL2
CL14
CL7
−log10(P−value)
10
5
0
−5
−10

Figure S2: Network of all protein interactions. Highly-confident interactions are in red others in blue.

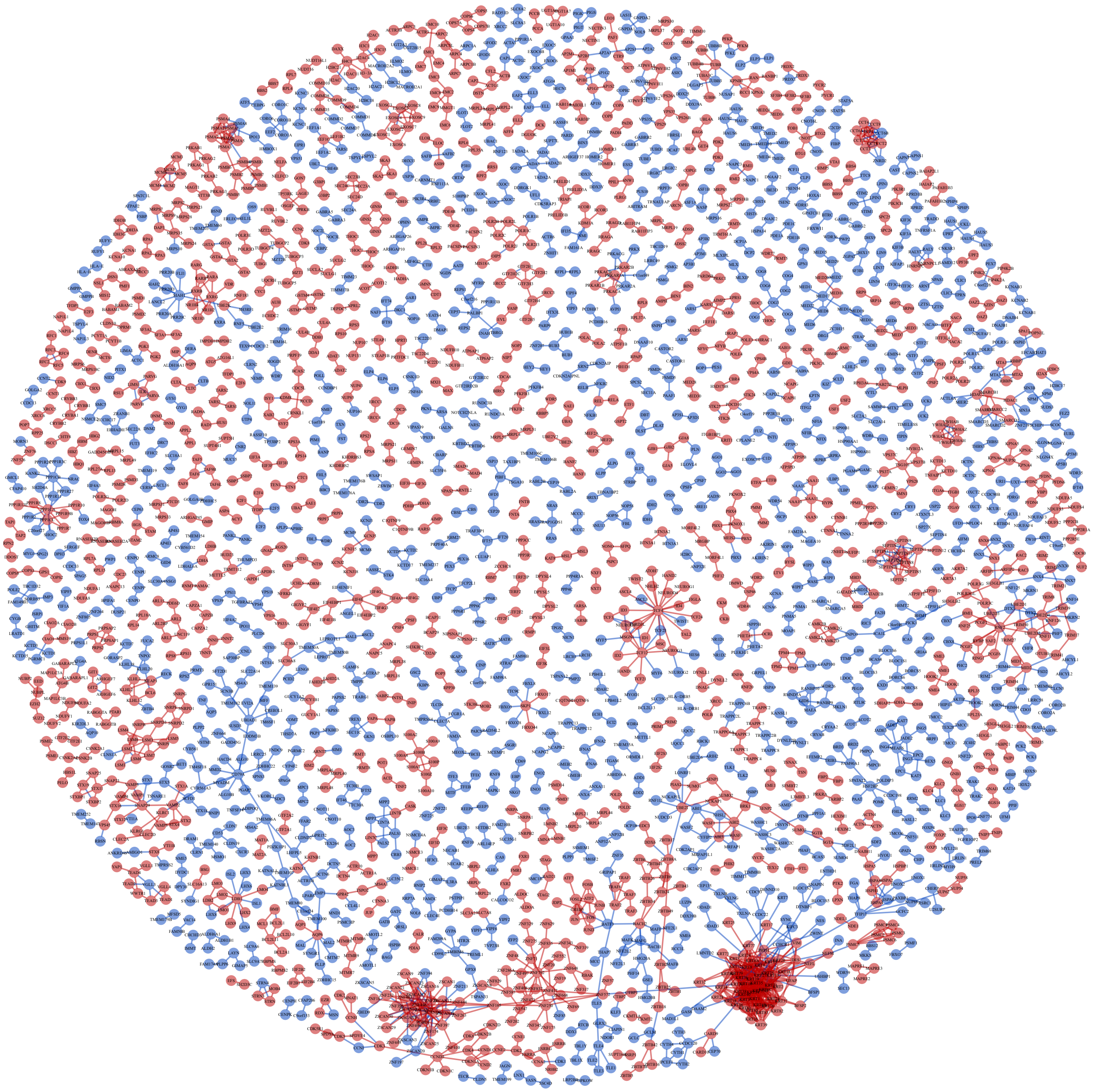

Figure S3: Modelling of the ERCC3 complex.

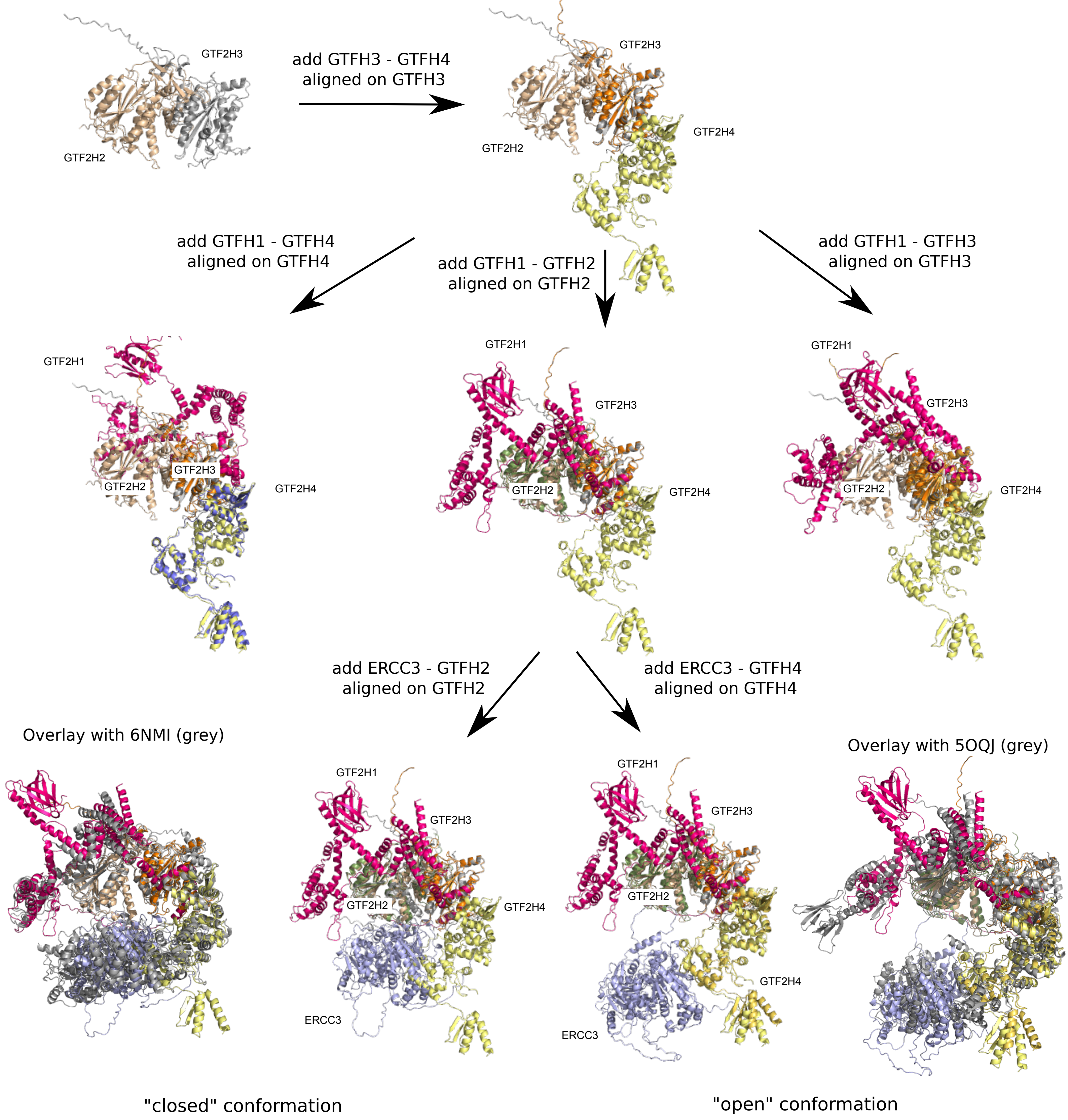

GTF2H3
GTF2H2
add GTFH3 - GTFH4
aligned on GTFH3
GTF2H3
GTF2H4
GTF2H2
add GTFH1 - GTFH4
aligned on GTFH4
add GTFH1 - GTFH2
aligned on GTFH2
add GTFH1 - GTFH3
aligned on GTFH3
GTF2H1
GTF2H3
GTF2H2
GTF2H4
GTF2H1
GTF2H3
GTF2H2
GTF2H4
GTF2H1
GTF2H3
GTF2H2
GTF2H4
add ERCC3 - GTFH2
aligned on GTFH2
add ERCC3 - GTFH4
aligned on GTFH4
Overlay with 6NMI (grey)
GTF2H1
GTF2H3
GTF2H2
GTF2H4
ERCC3
GTF2H1
GTF2H3
GTF2H2
GTF2H4
ERCC3
Overlay with 5OQJ (grey)
"closed" conformation
"open" conformation

Figure S4: Modelling of the large complexes Methanothermococcus thermolithotrophicus (pdb:5ODC) and the eukaryotic translation initiation factor 2B from Schizosaccharomyces pombe (pdb:5B04).

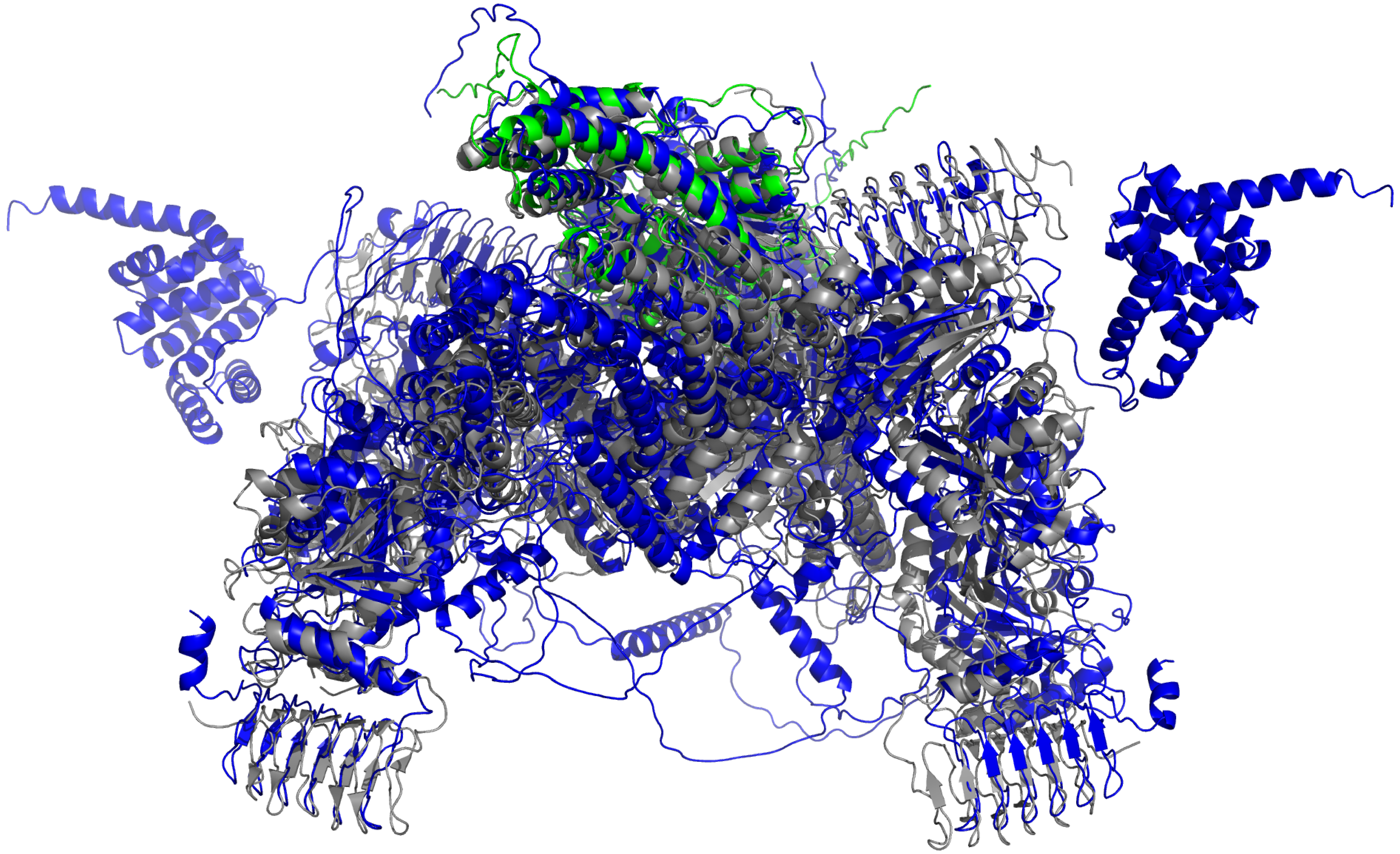

5B04

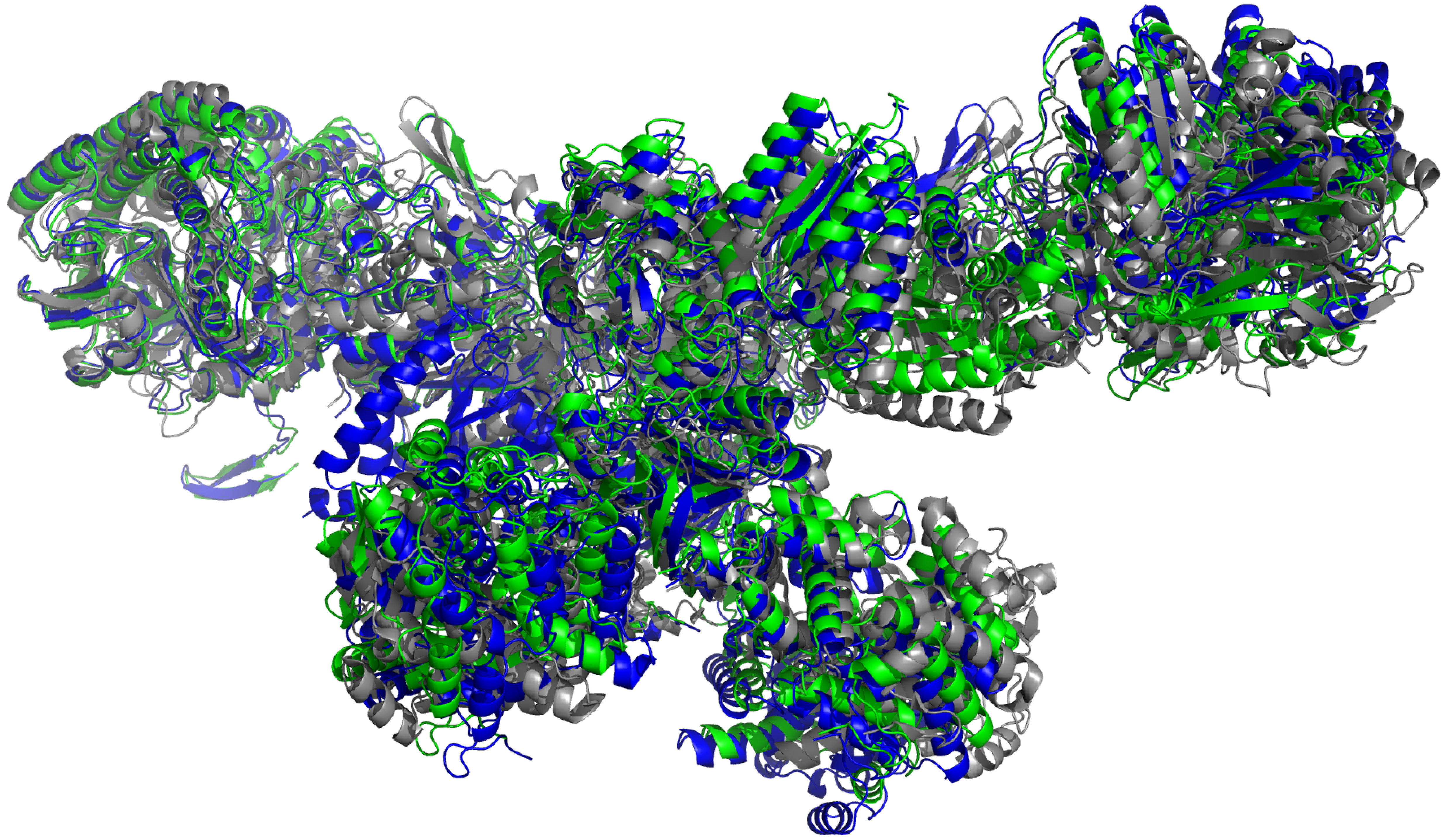

5ODC

Grey: PDB structure, Green: Model build from dimers, Blue: Model built from trimers
